# T1-11, an adenosine derivative, ameliorates aging-related behavioral physiology and senescence markers in aging mice

**DOI:** 10.18632/aging.103279

**Published:** 2020-06-05

**Authors:** Wei-Hsiang Hsu, Young-Ji Shiao, Yen-Ming Chao, Yi-Jeng Huang, Yun-Lian Lin

**Affiliations:** 1 Department of Chinese Pharmaceutical Sciences and Chinese Medicine Resources, China Medical University, Taichung 40402, Taiwan; 2 National Research Institute of Chinese Medicine, Ministry of Health and Welfare, Taipei 11221, Taiwan; 3 Institute of Biopharmaceutical Science, National Yang-Ming University, Taipei 112, Taiwan; 4 Department of Pharmacy, National Taiwan University, Taipei 10050, Taiwan

**Keywords:** T1-11, adenosine analog, senescence, neurogenesis, anti-neuroinflammation

## Abstract

Aging is a natural human process. It is uniquely individual, taking into account experiences, lifestyle habits and environmental factors. However, many disorders and syndromes, such as osteoporosis, neurodegenerative disorders, cognitive decline etc., often come with aging. The present study was designed to investigate the possible anti-aging effect of *N^6^*-(4-hydroxybenzyl)adenine riboside (T1-11), an adenosine analog isolated from *Gastrodia elata*, in a mouse model of aging created by D-galactose (D-gal) and the underlying mechanism, as well as explore the role of adenosine signaling in aging. T1-11 activated A_2A_R and suppressed D-gal- and BeSO_4_-induced cellular senescence *in vitro*. *In vivo* results in mice revealed that T1-11 abated D-gal-induced reactive oxygen species generation and ameliorated cognitive decline by inducing neurogenesis and lowering D-gal-caused neuron death. T1-11 could be a potent agent for postponing senility and preventing aging-related neuroinflammation and neurodegeneration.

## INTRODUCTION

Aging is a progressive, accumulative and natural phenomenon, associated with multiple and irreversible physiological and pathological changes, including the formation of advanced glycation end-products (AGEs) and free radical damage in cells, tissues and organs of an organism [1]. It is responsible for the pathogenesis of age-related disorders or syndromes including insomnia, osteoporosis, atherosclerosis and cardiovascular diseases, liver and kidney failure and immune system dysfunction as well as neuro-degenerative disorders, especially Alzheimer disease (AD) and Parkinson disease (PD) [2]. Aging is also associated with cognitive deterioration, including memory loss and impaired induction of long-term potentiation [3].

Several chemical agents, such as beryllium salts and D-galactose (D-gal), have been reported to cause cellular senescence [4, 5] and syndromes similar to aging [6, 7]. Cellular senescence was originally identified as a stable exit from the cell cycle caused by the finite proliferative capacity of cells. Currently, senescence is considered to be caused by oxidative stress that can be induced by a wide range of intrinsic and extrinsic insults, including oncogenic activation, oxidative and genotoxic stress, and mitochondrial dysfunction [8]. All *in vivo* biomarkers for senescence, including increased senescence-associated β-galactosidase (SA-β-gal) activity and p16^INK4a^ expression, activation of the p53 and/or p21^CIP1^ pathway, senescence-associated secretory phenotype-related cytokine secretion, and telomere shortening [8]. Beryllium salts could inhibit cell division and induce premature senescence at low micromolar concentration [4]. D-gal is one kind of hexoses and can be transported into cells via glucose transporters and converted to glucose via enzymatic reactions. Then, D-gal could be converted to galactitol by aldose reductase. However, galactitol cannot be metabolized; its accumulation often results in osmotic imbalance and then harmful oxidative stress in cells [6, 7]. As a result, increased oxidative stress causes aging-like changes, such as upregulation of both p53 and p21 in brain, liver, kidney, etc. Thus, D-gal has been used to induce aging in mice for aging pharmacological research [5]. Further, D-gal-induced brain aging processes in mice resemble those in humans [5, 6]. The major molecular mechanisms of D-gal-induced brain aging involve the production of reactive oxygen species (ROS) and subsequent ROS-induced mitochondrial dysfunction [5, 9]. Moreover, a high dosage of D-gal inhibits the expression of nerve growth factors and their associated proteins, which results in the degeneration of neurons and further impairment of long-term potentiation and neurogenesis in the hippocampus, thereby causing memory dysfunction [5].

*N^6^*-(4-hydroxybenzyl)adenine riboside)) (designated T1-11), an adenosine analog with the activities of an agonist of A_2A_ adenosine receptor (A_2A_R) and an inhibitor of equilibrative nucleoside transporter 1 (ENT1, adenosine transporter), was isolated from the tuber of *Gastrodia elata* (Tianma) [10]. Receptor binding assays showed T1-11 could activate adenosine 2A (A_2A_R) (IC_50_= 4.66 μM; Ki= 2.62μM; agonist) and A_3_ receptors (IC_50_= 0.11μM; Ki= 0.10μM; not significant in function), inhibit ENT1 (IC_50_= 1.57μM; Ki= 0.54μM; inhibitor), ameliorate motor degeneration in a mouse model of Huntington disease (HD) [11], and extend the lifespan of a mouse model of Niemann-Pick type C disease [11, 12]. There are several types of adenosine receptors such as A_1_, A_2A_R, and A_3_, and are widely distributed in different brain areas, including striatum, hippocampus, cerebral cortex etc., for modulating the release of neurotransmitters and controlling cognition and motivational responses [13]. Inhibition of adenosine transporter blocks adenosine re-uptake and recycles adenosine from the extracellular space, which causes elevated adenosinergic tone in brain and plays a significant role in neuronal and cognitive changes during natural aging [14] and improves memory impairment in APP/PS1 mice [15]. Castillo et al. demonstrated lower level of A_2A_R in the senescence-accelerated prone mouse model (SAMP8) [16]. The activation of A_2A_R enhances nerve growth factor (NGF)-induced neurite outgrowth in PC12 cells and rescues NGF-induced neurite outgrowth impaired by blockade of the mitogen-activated protein kinase cascade [17], which may improve neuron loss during aging. These studies suggest that adenosine augmentation and A_2A_R activation may improve aging-related syndromes or disease.

In this study, we investigated the effect of T1-11 in D-gal-and BeSO_4_-induced SH-SY5Y senescence cells and a D-gal-induced aging mouse model. We also examined the effect of T1-11 regulation on neurogenesis and neuron survival. Our study could provide important information for the beneficial effect of T1-11 on aging-related dementia as well as the role of adenosine augmentation in aging.

## RESULTS

### Effect of different concentrations of BeSO_4_ and D-gal in SH-SY5Y cells

The A_2A_R level was reduced in SH-SY5Y cells from passage 1 to 25 (supplementary Figure 1). Previous studies showed that BeSO_4_ and D-gal could trigger cell senescence [4, 9]. First, we used BeSO_4_ and D-gal to build a cell model for testing the effect of T1-11 on cell senescence and the role of A_2A_R in aging. SH-SY5Y cells were exposed to different concentrations of BeSO_4_ or D-gal, then cell viability was tested. Respectively, cells were treated with 15, 30, 60, and 90 μM BeSO_4_ for 24 hr, or 50, 100, 200, and 400 mM D-gal for 48 hr. Cell viability significantly decreased concentration-dependently (Supplementary Figure 2). Next, the senescence marker, SA-β-gal activity, was examined in SH-SY5Y cells after BeSO_4_ and D-gal treatment. Cellular senescence activity increased with a high concentration of BeSO_4_ and D-gal: 30 μM BeSO_4_ and 100 mM D-gal were effective concentrations with respect to cell viability (Supplementary Figure 2) and selected as the sublethal concentration for all subsequent experiments.

**Figure 1:**
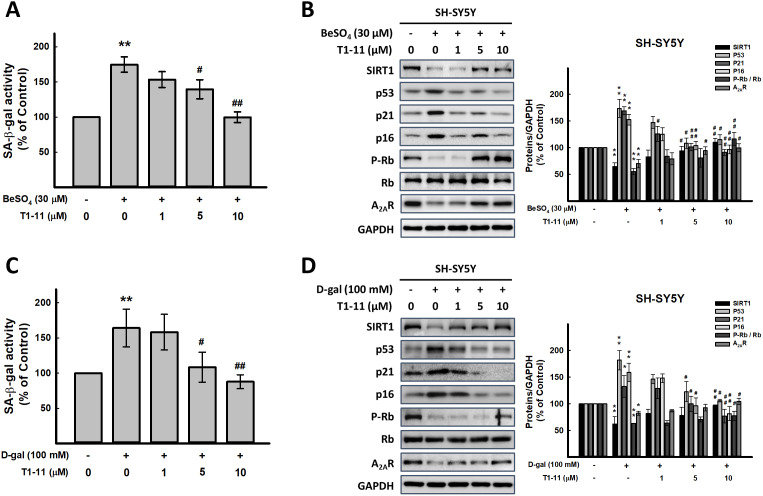
**Effect of T1-11 on cellular senescence after BeSO_4_- and D-gal-induced senescence.** (**A**, **C**) Effect of pre-treatment of T1-11 on BeSO_4_- and D-gal-induced cellular senescence markers, SA-β-gal activity, in SH-SY5Y cells. (**B**, **D**) Effect of pre-treatment of T1-11 on BeSO_4_- and D-gal-induced cellular senescence related molecules in SH-SY5Y cells. Data are mean±SEM from at least five independent experiments. Significant difference between control and treated cells is indicated by **p < 0.01. Significant difference between the cells treated with D-gal or BeSO_4_ alone and the cells treated with D-gal or BeSO_4_ combined with T1-11 is indicated by #*p* < 0.05, ##*p* < 0.01.

### T1-11 markedly diminished BeSO_4_- and D-gal-induced cellular senescence

Pretreating or cotreating cells with T1-11 (1-10 μM) or A_2A_R specific agonist, CGS21680, both significantly reduced cellular senescence marker, SA-β-gal activity (Figure 1A, 1C, and Supplementary Figures 3 and 4). T1-11 concentration-dependently reversed the A_2A_R level reduced by BeSO_4_ and D-gal (Figure 1B, 1D).

Because ROS generation is usually associated with aging, we assessed the antioxidant activity of T1-11. SH-SY5Y cells were pre-treated with T1-11 for 24 hr, then exposed to BeSO_4_. After 24 hr of incubation, SOD and CAT activities were assayed. SOD and CAT activities were significantly reduced with BeSO_4_ alone as compared with controls and increased concentration-dependently with T1-11 treatment (Supplementary Figure 5A, 5B).

Increased ROS levels would cause mitochondrial dysfunction, which plays an important role in brain aging or brain senescence and several aspects of aging-associated neurodegeneration [3]. Thus, we evaluated the effect of T1-11 on BeSO_4_-induced disruption of mitochondrial membrane potential (ΔΨm) by JC-1 staining. ΔΨm as seen by fluorescence emission shift was downregulated in SH-SY5Y cells treated with BeSO_4_ relative to controls, but preincubation with T1-11 reversed the BeSO_4_-decreased ΔΨm (Supplementary Figure 5C).

Activation of Arf-p53-p21 and p16-Rb pathways involve cellular senescence [8]. BeSO_4_ and D-gal elevated the levels of senescence-associated molecules p53, p21 and p16 and lowered the level of phospho-Rb (Figure 1B, 1D and Supplementary Figure 3B, 3D) were also found in our study. SIRT1, a type III protein deacetylase, is considered an anti-aging protein involved in regulating cellular senescence/aging, stress resistance, and inflammation [18]. We found lower SIRT1 level in BeSO_4_- and D-gal-induced aging cells, which was reversed by T1-11 pretreatment (Figure 1B, 1D and Supplementary Figure 3B, 3D).

### A_2A_R inhibition could trigger cellular senescence

**Figure 2:**
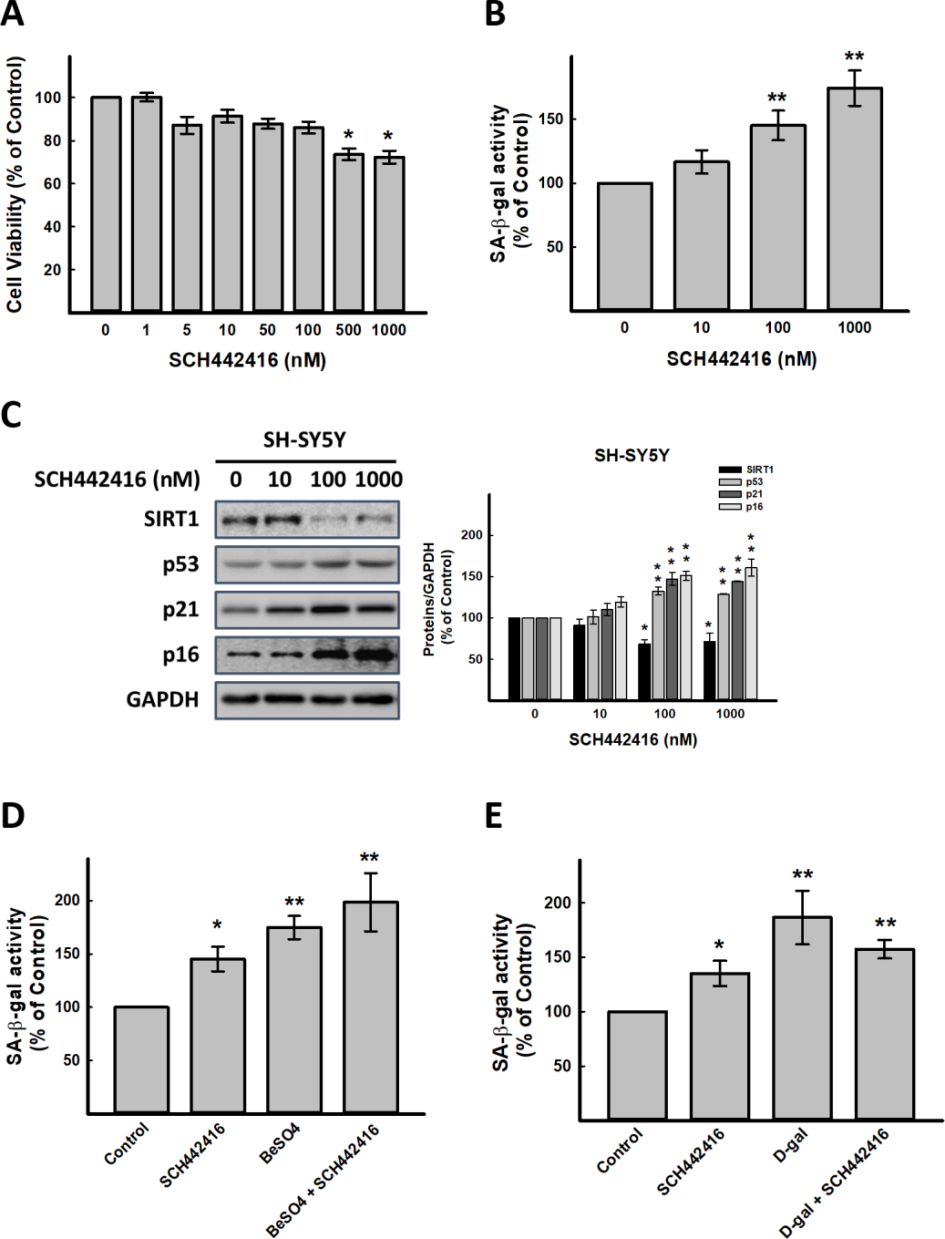
**Effect of A_2A_R antagonist SCH442416 on cellular senescence *in vitro.*** (**A**) Effect of SCH442416 on cell viability detected by SRB assay. Effect of SCH442416 on cellular senescence detected by (**B**) cellular senescence markers, SA-β-gal activity and (**C**) western blot analysis of SCH442416-induced cellular senescence related molecules. Combination test for (**D**) SCH442416 and BeSO_4_ as well as (**E**) SCH442416 and D-gal detected by cellular senescence markers, SA-β-gal activity. Data are mean±SEM from at least four independent experiments. Significant difference between control and treated cells is indicated by *p < 0.05, **p < 0.01.

T1-11 is an agonist of A_2A_R. Next, we used SCH442416, a highly selective A_2A_R antagonist [19], was used to explore whether A_2A_R signaling inhibition affects on aging progress. As illustrated in Figure 2A, SCH442416 at 0.5 and 1 μM significantly decreased cell viability. SCH442416 enhanced SA-β-gal activity in SH-SY5Y cells in a concentration-dependent manner (*p* < 0.05, Figure 2B), and increased cellular senescence-associated molecules, p53, p21 and p16 in a concentration-dependent manner as well (Figure 2C). Besides, A_2A_R antagonists (SCH442416 or SCH58261) could abrogate the actions of T1-11 (Supplementary Figure 6). Combining the results of SCH442416 and BeSO_4_ and D-gal in SH-SY5Y cells exhibited that these agents have no synergistic effects on aging progress (Figure 2D, 2E).

Using LNA-A_2A_R induced A_2A_R silence to confirm that A_2A_R is essential for aging progress. The data demonstrated the similar results with SCH442416, but we found that LNA-A_2A_R could enhanced BeSO_4_ action (Supplementary Figure 7). This result might display that expression of A_2A_R could affect aging progress. Integrated the results of Figures 1, 2 and Supplementary Figures 1–7, indicated that A_2A_R might play an important role in retard aging.

### T1-11 did not induce side-effect in D-gal-treated mice

D-gal-induced aging model in mice has been widely used for anti-aging medicine research [7]. The aging degree is close to 16-24 months in mice, and all these changes are consistent with natural aging [6].

During the 8-week period of our experiment, the daily appearance and behavioral activities of mice were recorded weekly; mice showed no pathological changes, such as vomiting, and no inflammation in injection sites of mice in any group. Moreover, mouse groups did not differ in food intake and body weight change (*p*>0.05) (Supplementary Figure 8). Mice with D-gal treatment showed the symptoms of aging, including memory loss and slow movement (Figures 4 and 5).

### Effect of T1-11 on serum parameters in D-gal-treated mice

D-gal markedly increased the serum levels of blood glucose, blood urea nitrogen (BUN), lactate dehydrogenase (LDH), aspartate transaminase (AST), alanine transaminase (ALT), triglyceride (TG) and total cholesterol (TC) (Supplementary Table 1). T1-11 treatment at 10 mg/ml significantly reversed all of these indexes. By vitamin E (Vit.E), Vit.E only decreased serum GOT and LDH levels. Thus, T1-11 is better than Vit.E to attenuate aging parameters in blood.

### T1-11 inhibited D-gal-induced oxidative stress by elevating multiple antioxidative enzymes in mice

**Figure 3:**
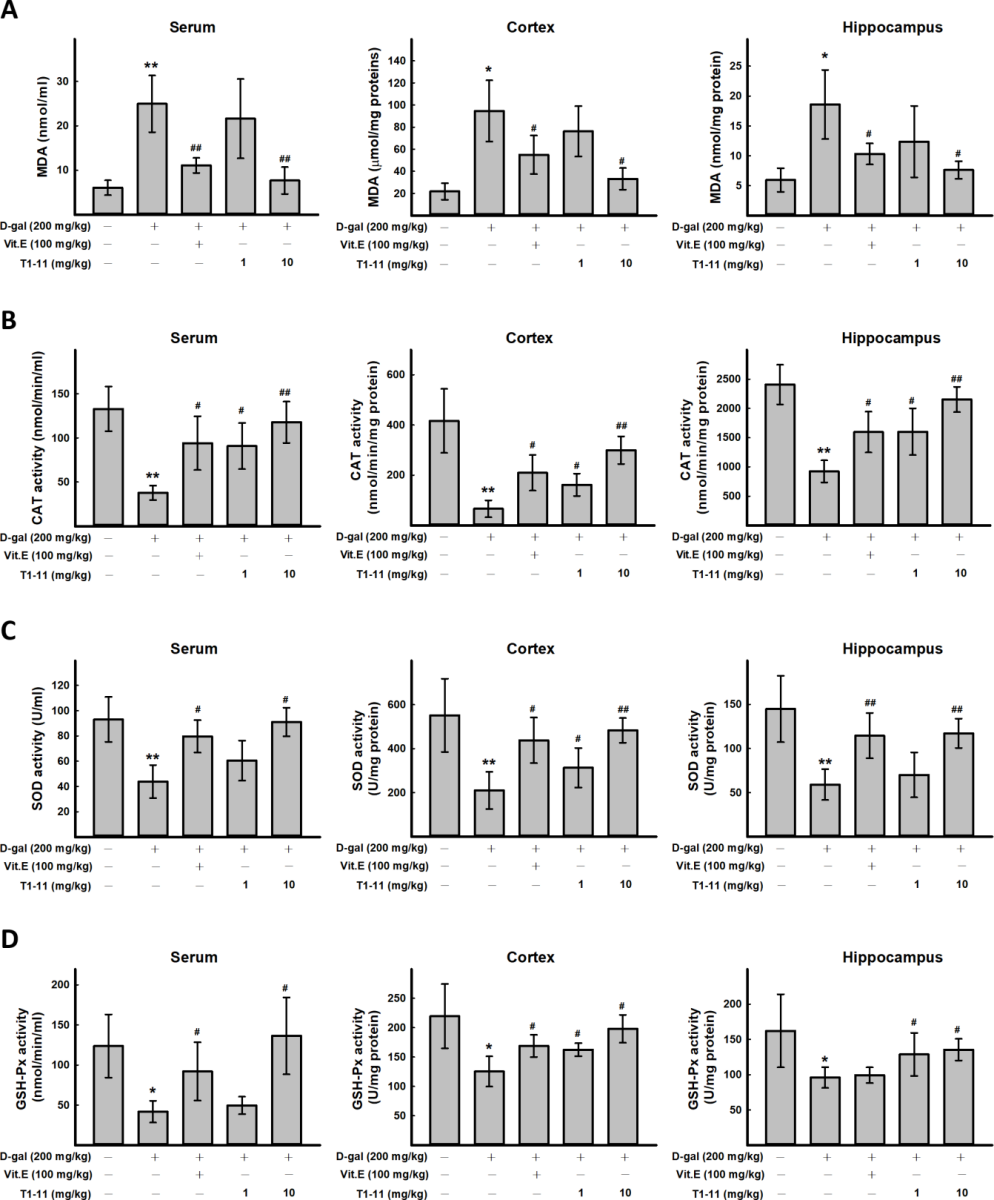
**The effect of T1-11 on oxidant/antioxidant stress parameters in the serum, cortex, and hippocampus of D-gal-induced aging mice.** After treament, serum, cortex, and hippocampus tissue of were collected. The level of MDA (**A**) and the activity of CAT (**B**), SOD (**C**), and GSH-Px (**D**) in the serum, cortex, and hippocampus of D-gal-induced aging mice were detected. Data are mean ± SD (n = 6). Significant difference between control and D-gal-induced aging mice is indicated by * *p* < 0.05, ** *p* < 0.01 compared with the control group. Significant difference between the mice treated with D-gal alone and the mice treated with D-gal combined with Vit.E or T1-11 is indicated by #*p* < 0.05, ##*p* < 0.01 compared with D-gal group.

ROS plays an important role in aging. ROS production is one of the most important mechanisms underlying D-gal-induced aging [6]. In this study, the content of the oxidative stress marker malondialdehyde (MDA) and activities of antioxidative defense enzymes superoxide dismutase (SOD), catalase (CAT), and glutathione peroxidase (GSH-Px) in the cortex and hippocampal tissue and serum of mice as biomarkers of oxidative stress in D-gal-induced aging were measured. We found D-gal treatment greatly increased MDA level in serum, cortex, and hippocampus, and T1-11 significantly reversed the D-gal-induced MDA level (Figure 3A). Thus, the lipid peroxide process in mice was accelerated by D-gal, and T1-11 and Vit.E were effective in reducing MDA content in both brain tissue and serum. D-gal significantly decreased SOD, CAT and GSH-Px activities as compared with controls (Figure 3B–3D), which were markedly elevated with T1-11 and Vit.E treatments.

### T1-11 recovered the cognitive decline in D-gal-induced aging mice

**Figure 4:**
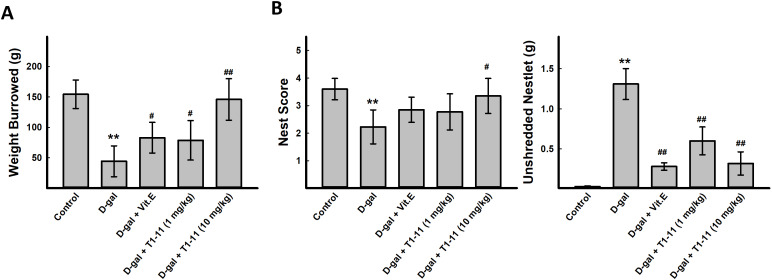
**T1-11 ameliorates behavior deficits in D-gal-induced aging mice.** The tasks of burrowing (**A**) and nesting (**B**) were performed after treatment. Data are mean±SEM (n = 6). Significant difference between control and D-gal-induced aging mice is indicated by * *p* < 0.05, ** *p* < 0.01 compared with the control group. Significant difference between the mice treated with D-β-gal alone and the mice treated with D-gal combined with Vit.E or T1-11 is indicated by #*p* < 0.05, ##*p* < 0.01 compared with D-gal group. 1: Control; 2: D-gal (200 mg/kg); 3: D-gal (200 mg/kg) + Vit.E (100 mg/kg) (positive control); 4: D-gal (200 mg/kg) + T1-11 (1 mg/kg); 5: D-gal (200 mg/kg) + T1-11 (10 mg/kg).

Cognitive behavioral deficit is one of the aging-related markers of brain dysfunction. A variety of species-specific spontaneous behaviors, including burrowing and nesting behaviors, which engage a broad network of brain regions, have previously been applied on evaluating the activities of daily living (ADL) skills of aging mice. Burrowing and nesting behaviors were examined. D-gal-induced aging mice showed deficits in spontaneous burrowing and nesting behaviors, which were significantly ameliorated by Vit.E treatment (100 mg/kg) and T1-11 at both 1 and 10 mg/kg in burrowing (Figure 4A) and the nest score and unshredded nestlet (Figure 4B).

### Effect of T1-11 on D-gal-induced spatial memory decline

**Figure 5:**
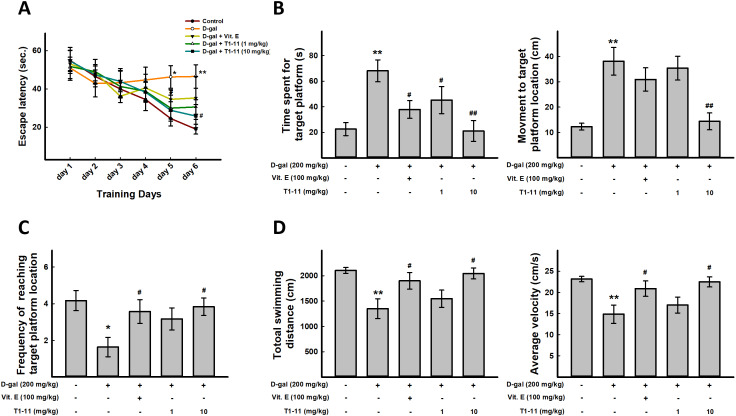
**T1-11 improves spatial learning, impaired memory, and sport activity decline in D-gal–induced aging mice.** For behavioral study, N = 6 mice per group were used. Morris water maze was performed. (**A**) Mean escape latency time (sec) to reach hidden platform during training session. Time spent in the target quadrant (where the platform was located during the hidden platform training session) during the probe test (**B**) and number of platform crossings over the previous platform place during the probe test (**C**) were examined. (**D**) Total swimming distance and average of swimming speed were examined. Data are mean±SEM (n = 6). Significant difference between control and D-gal-induced aging mice is indicated by * *p* < 0.05, ** *p* < 0.01 compared with the control group. Significant difference between the mice treated with D-gal alone and the mice treated with D-gal combined with Vit.E or T1-11 is indicated by #*p* < 0.05, ##*p* < 0.01 compared with D-gal group.

Morris water maze task was used to evaluate the spatial learning and memory ability of mice. Escape latency was calculated by the time taken for the mice to reach the hidden platform. Mice with impaired memory and learning should travel longer before they can arrive at the set platform, thereby taking more time to find the platform. The escape latency was longer for D-gal-treated mice than control mice during the training period, which indicates poorer learning and memory performance. T1-11 treatment of D-gal-treated animals significantly decreased escape latency as compared with D-gal treatment alone on each training days (Figure 5A).

After 6 days of training, the probe trial was performed. D-gal-treated mice spent more time to find the target platform location as compared with controls, but less time in T1-11- and Vit.E-treated mice (Figure 5B). In addition, T1-11-and Vit.E-treated mice crossed the platform location more often than with D-gal alone (Figure 5C). These data suggest that T1-11 improves D-gal-induced spatial learning and memory impairment.

### Effect of T1-11 on D-gal-induced sport activity decline

Swimming distance and average swimming speed were captured from the automated software, based on the total distance that a mice travels at the probe trial period. We found that D-gal-induced aging mice have decline sport activity and slow movement. T1-11 and Vit.E treatment could meliorate these aging characteristics (Figure 5D).

### Effect of T1-11 on neurodegeneration in the hippocampus

**Figure 6:**
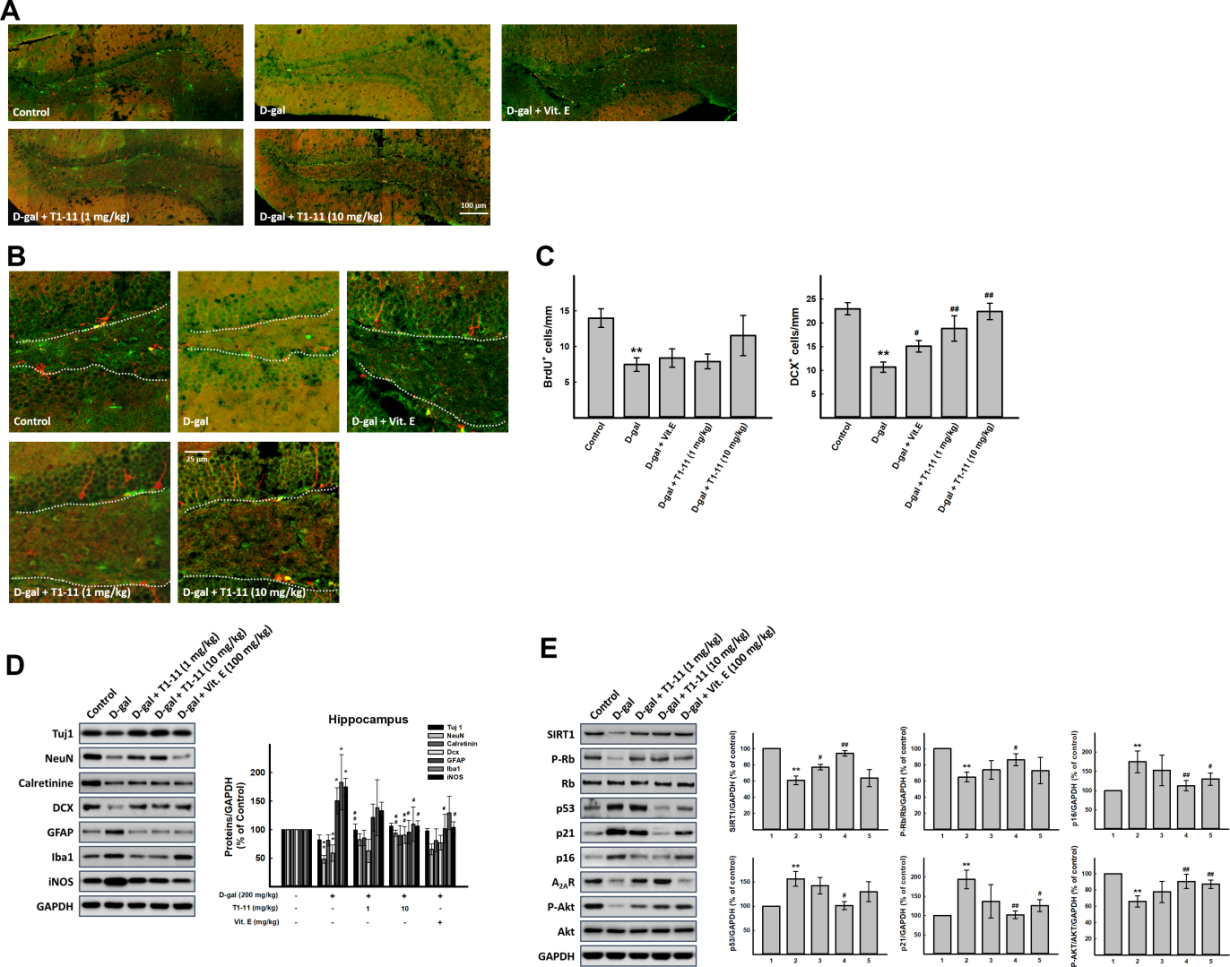
**Effect of T1-11 on neurogenesis markers in hippocampus of D-gal-induced aging mice.** Hippocampal neurogenesis was detected by immunohistochemical staining with double cortin (DCX, red) and BrdU antibody (green). The full representative immunostaining images and partial enlargement of the dentate gyrus (DG) area are shown in (**A**, **B**). (**C**) The number/mm SGZ of BrdU positive cells (BrdU^+^) and DCX positive cells (DCX^+^) are counted. Western blot analysis of (**D**) neuron aging related molecules and (**E**) cellular senescence markers. Data are mean±SEM (n = 6). Significant difference between control and D-gal-induced aging mice is indicated by * *p* < 0.05, ** *p* < 0.01 compared with the control group. Significant difference between the mice treated with D-gal alone and the mice treated with D-gal combined with Vit.E or T1-11 is indicated by #*p* < 0.05, ##*p* < 0.01 compared with D-gal group. 1: Control; 2: D-gal (200 mg/kg); 3: D-gal (200 mg/kg) + Vit.E (100 mg/kg) (positive control); 4: D-galactose (200 mg/kg) + T1-11 (1 mg/kg); 5: D-galactose (200 mg/kg) + T1-11 (10 mg/kg).

Aging-related hippocampal dysfunction, such as cognitive and memory deficits, is characterized by compromised neuronal plasticity, decreased neurogenesis, and neuronal death [1, 20]. Hippocampal neurogenesis and brain plasticity can be improved by dietary factors, leading to possible improvement in age-related cognitive deficits [21]. Thus, to estimate the rate of hippocampal neurogenesis during aging, we counted 5-bromo-2′-deoxyuridine (BrdU) nuclei and doublecortin (DCX^+^) cell number in the hippocampal dentate gyrus of D-gal-induced aging mice. BrdU^+^ nuclei of proliferating cells are mainly detected in the subgranular zone (SGZ) of the dentate gyrus (Figure 6A, 6B). The number of BrdU^+^ nuclei was markedly reduced in the hippocampus of D-gal–treated mice. Notably, the number of BrdU^+^ nuclei was increased with T1-11 and Vit.E treatment but not significantly as compared with D-gal alone (Figure 6A–6C).

In control mice, DCX^+^ neuroblasts and immature neurons were observed in the SGZ, with well-developed dendrites extending into the molecular layer of the dentate gyrus (Figure 6A, 6B). DCX is a microtubule-associated protein expressed in neural stem cells and immature neurons and thus can be used to visualize migrating neuroblasts. After chronic treatment with D-gal, the number of DCX^+^ neuroblasts was significantly decreased, and these neuroblasts exhibited poorly developed dendrites with D-gal alone (Figure 6A–6C). However, the reduction was markedly attenuated by T1-11 and Vit.E treatment (Figure 6A–6C). The histological results were further confirmed by Western blot analysis. The findings by Western blotting analysis are in line with those by histological staining. The protein expression of DCX was significantly decreased in the hippocampus in the D-gal alone group, whereas the reduction was effectively attenuated by treatment with T1-11 and Vit.E (Figure 6D). Moreover, T1-11 treatment also attenuated D-gal-reduced NeuN expression in mature neurons in the hippocampus (Figure 6D). Additionally, D-gal treatment slightly decreased the expression of neuron-specific class III β-tubulin (Tuj1). T1-11 and Vit.E treatment reversed this reduction to the level of control group. These findings may suggest that T1-11 improves cognitive function by ameliorating D-gal-suppressed hippocampal neurogenesis.

The PI3K/Akt signaling pathway is one of the most important pathways in cell proliferation and differentiation and also a pivotal function in mediating cell survival. It also inhibits apoptosis, maintains stem cell pluripotency and regulates neurogenesis in the SGZ of the hippocampus [22]. Akt is the signaling molecule downstream of PI3K and participates in regulating many biological processes, including neurogenesis, cell proliferation, growth and apoptosis [23]. The phospho-Akt/Akt ratio was lower in the hippocampus of D-gal-treated mice than controls, and T1-11 and Vit.E treatment markedly elevated the phosphorylation of Akt (Figure 6E). Hence, T1-11 could activate the PI3K/Akt pathway to promote neurogenesis or prohibit neuronal death in the mouse hippocampus.

### T1-11 ameliorated the inflammation in the hippocampus

Recent evidence showed that neuroinflammation contributes to cognitive dysfunction in pathological conditions [24]. Activated microglia and astrocytes can have adverse effects on neurogenesis by producing ROS or releasing inflammatory cytokines and are being considered a pathological hallmark in neurodegenerative disorders.

Therefore, we investigated the status of astrocytes and microglia. Glial fibrillary acidic protein (GFAP) and anti-ionized calcium binding adapter molecule 1 (Iba1) are specific markers of activated astrocytes and microglia cells, respectively. Chronic administration of D-gal activated astrocytes and increase the expression of GFAP in the hippocampus [25]. These results agree with our Western blot analysis results. The expression of GFAP, Iba1, and induced nitric oxide synthase (iNOS) in the D-gal group was upregulated significantly as compared with controls, so astrocytes and microglia were activated in D-gal-induced aging mice; this activation was decreased by T1-11 treatment (Figure 6D). In addition, we also found that levels of TNF-α, which is one of inflammation markers, were higher in hippocampus of D-gal-induced aging mice, and T1-11 treatment could ameliorate it (Supplementary Figure 9).

### Effect of T1-11 on expression of aging-related markers in the hippocampus of D-gal-induced aging mice

To confirm the effect of T1-11 on preventing cellular senescence, we detected the expression of known aging-related markers, SIRT1, p16, p53, and p21, in the hippocampus. D-gal treatment significantly increased p16, p53, and p21 expressions and decreased SIRT1 expression in the hippocampus as compared with controls. T1-11 reversed D-gal-induced p16, p53, and p21, and SIRT1 expressions (Figure 6E). Besides, T1-11 could decrease D-gal-induced apoptosis in hippocampus of our aging mice (Supplementary Figure 10).

## DISCUSSION

Previous study indicated that the level of A_2A_R was lower in the senescence-accelerated prone mouse model (SAMP8) as compared with the senescence-accelerated resistant mouse model (SAMR1) [16]. As well, activation of A_2A_R inhibits senescence by downregulating p53 [26]. We also found that A_2A_R expression was slightly reduced in older cells. However, the role of A_2A_R in aging is not yet fully elucidated previously. The present study showed that (1) A_2A_R inhibition could induce premature senescence and the expression of cellular senescence markers in SH-SY5Y; (2) T1-11 suppressed the cellular senescence of SH-SY5Y; (3) T1-11 ameliorated D-gal-induced cognitive decline in mice; and (4) T1-11 treatment elicited neurogenesis as well as reduced neuroinflammation and the expression of cellular senescence markers in the hippocampus of D-gal-induced aging mice.

Aging has a great impact on brain functions. Older people have decreased memory, including recognition memory, short-term recall, long-term memory and speed of processing [1]. Some natural foods or herbal medicine have been found to benefit in with anti-aging activities and as potential candidates for anti-aging therapies. *G. elata* is one of the Chinese medicinal used for treating neurasthenia, insomnia, dizziness, epilepsy, and nervous headache [27]. Currently, more than 81 compounds, including gastrodin, vanillin, gastrodigenin and parishins, were isolated and identified from this plant [27]. T1-11 is one of compounds from *G. elata*, which was found to prevent apoptosis in serum-deprived PC12 cells by suppressing JNK activity [10, 28]. We also found that T1-11 could decrease D-gal-induced cell death (Supplementary Figure 1E). In addition, T1-11 was found a multiple-action compound with an A_2A_R agonist and ENT1 inhibitor [28], which had beneficial effects on neurodegenerative diseases such as HD [11]. Furthermore, intraperitoneal injection of T1-11 increased the cAMP level in the brain of wild-type mice but not A_2A_R-knockout mice [11]. These findings indicate that T1-11 acts as an A_2A_R agonist and ENT1 inhibitor could activate adenosinergic tone in synapses, where the adenosine receptor and transporter are located, and alleviate neurodegeneration. However, the lower A_2A_R (IC_50_= 4.66 μM; Ki= 2.62 μM) and ENT1 (IC_50_= 1.57 μM; Ki= 0.54 μM) affinities of T1-11 indicated the effect of T1-11 may not directly activate A_2A_R. ENT1 is one of major role in brain for adenosine uptake and present in many brain areas, including hippocampus and cortex [29] with high efficiency in adenosine re-uptake. Therefore, the adenosine augmentation may play an important role in the effect of T1-11.

During neuronal development, A_2A_R expression is transiently regulated in various areas of the developing rat brain [30]. Furthermore, stimulation of A_2A_R activates at least two major cellular signaling cascades: adenylyl cyclase/cAMP/protein kinase A (PKA) and protein kinase C (PKC)-mediated pathways [31]. A_2A_R stimulation, with adenosine or pharmacological agonists, activates G protein and consequently adenylate cyclase and PKA. PKA feedbacks to phosphorylate A_2A_R and shifts its coupling to Gi protein and Src kinase, thus activating the surviving mediator PI3K and its downstream effector AKT to promote cell survival [32]. Activation of A_2A_R increased cAMP level and triggered a canonical Wnt pathway, which could regulate neurogenesis and regeneration processes [31, 33]. A similar A_2A_R-mediated neuroprotection mechanism has been shown to occur in hippocampal neurons after brain derived neurotrophic factor (BDNF) withdrawal [32]. Additionally, translin-associated protein X and kinesin-like protein 2A could directly interact with A_2A_R by binding to the C terminus of A_2A_R and triggering the PI3K/AKT pathway to rescue impaired neuronal differentiation and damaged neurogenesis [34, 35]. Our *in vivo* data illustrated that an A_2A_R agonist, T1-11, might promote neurogenesis or produce survival signaling through the AKT pathway in the hippocampus to ameliorate D-gal-induced memory loss in aging. Moreover, combination of A_2A_R specific antagonist, SCH58261, and T1-11, we found that SCH58261 impaired T1-11 activity for amelioration of D-gal-induced memory loss (data not shown).

D-gal can mimic aging in animal models by inducing oxidative stress and inflammation, and this models has been widely used in anti-aging research. Chronic administration of D-gal leads to a significant increase of free radicals and reacts with the amino group of peptides and proteins that form AGEs. AGEs bound to their receptors (RAGEs) is a common thread in aging, neuroinflammation, and neurodegeneration [7, 25]. Furthermore, D-gal could decrease the expression of memory-related proteins, deterioration of learning and memory function, and pathological alterations of astrocytes, which might be associated with the increased the expressions of inflammation-related genes such as IL-1β, TNF-α, and iNOS in the animal hippocampus [25]. D-gal-induced neuroinflammation causes microglia and astrocyte activation in the brain. A_2A_R has an immunoregulatory effect to prevent overly exuberant immune responses that can lead to collateral tissue damage [36]. Scheibner et al. reported severe overwhelming inflammation and tissue destruction in A_2A_R gene knockout mice [37]. A_2A_R activation confers beneficial anti-inflammatory effects and attenuates oxidative damage by inhibiting elevated ROS in inflammation [36]. A_2A_R agonists appear to inhibit inflammation by attenuating release of proinflammatory cytokines such as TNF-α and IL-12 release [38]. In addition, A_2A_R localizes in microglial cells and may imply a regulation of microglial function in response to brain damage [39]. Neuroinflammatory blockade is considered to enhance neural stem/progenitor cell activity and promote adult neurogenesis [40]. Our study also found that T1-11 suppressed D-gal-induced neuroinflammation and ROS generation and led to reduced neurogenesis or neuron loss in hippocampus, which boosted cognitive decline.

We found that A_2A_R activation and ENT1 inhibitor could improve neurodegeneration in aging. Our results are complementary to Moscoso-Castro et al., who found that deletion of A_2A_R led to deficits in spatial memory and learning as well as decreased neurogenesis [41]. However, A_2A_R activation or inhibition as a benefit for anti-aging is controversial [14, 42]. The hippocampal neurogenic niche is complex, and many types of cells interact to maintain a functional niche environment. Apart from neural stem and neuronal lineage cells, many niche-resident cells, such as microglia and astrocytes, have immunological characteristics that in the steady state, can regulate adult neurogenesis [43]. The present study only investigated the relation between A_2A_R and aging; whether. The other receptors or molecules might play a role in regulating aging by the other models of senility remains to be explored. In the future, we also need to have more evidence to explain how A_2A_R regulates neurogenesis and improves cognitive decline.

In summary, our *in vitro* data demonstrated that T1-11 suppressed D-gal- and BeSO_4_-induced cellular senescence, and A_2A_R inhibition triggered cellular senescence. *In vivo* results showed that T1-11 abated D-gal-induced ROS generation and also ameliorated cognitive decline via neurogenesis induction or lowering D-gal-caused neuron death. T1-11 also has neuroprotective effects against D-gal-induced memory dysfunction, neuroinflammation, and neurodegeneration. Adenosine augmentation could play an important role in aging and T1-11, as a potent anti-aging agent and prevent aging-related neuroinflammation and neurodegeneration.

## MATERIALS AND METHODS

### Drugs

The isolation and synthesis of *N^6^*-(4-hydroxybenzyl)-adenine riboside (defined as T1-11) were previously described [10, 28]. D-galactose, BeSO_4_, SCH442416, SCH58261, CGS 21680, and vitamin E were purchased from Sigma-Aldrich.

### Cell culture

The SH-SY5Y human neuroblastoma cell line was obtained from the Bioresource Collection and Research Center (BCRC) (Hsinchu, Taiwan). From our preliminary experiments, we used cells at passages 3-10 and 20-30 as young and senescent cells, respectively. Cells were cultured in Dulbecco’s modified Eagle’s medium (Hyclone) supplemented with 10% (v/v) fetal bovine serum, 0.05 U/ml penicillin, and 0.05 mg/ml streptomycin and maintained at 37 °C with 95% humidified air and 5% CO_2_. Cells in culture dishes were used for experiments after reaching 80% confluence.

### Sulforhodamine B (SRB) and MTT assays for cell viability

Cells were seeded into plates and cultured overnight at 37 °C in a 5% CO_2_ incubator, then treated with drugs. The medium was removed immediately after the drug treatment and replaced with 10% precooled trichloroacetic acid (TCA). The cells were fixed for 1 hr at 4 °C, then washed with distilled water and dried. Briefly, 100 μl of a 4-mg/mL solution of SRB (Sigma-Aldrich, St. Louis, MO, USA) in 1% acetic acid was added to each well and incubated for 1 hr. The plate was then washed five times with 1% acetic acid solution and dried. The SRB in cells was dissolved in 150 μL of 10 mM Tris-HCl and measured at 540 nm by using an ELISA reader. MTT assay was performed as usual method.

### SA-β-gal assay

The activity of senescence-associated β-galactosidase (SA-β-gal) is the most-used biomarker for senescence. Cultured aging cells were plated into 12-well dishes before staining. Cells were fixed and stained as the manufacturer’s protocol SA-β-gal staining kit (Cell Signaling #9860). Stained cells were viewed under a Leica DM IL LED microscope.

Cellular SA-β-gal activity was evaluated by using a 96-well cellular senescence assay kit (Enzo Life Sciences, Inc., Farmingdale, NY, USA), following the described assay protocol. The fluorescence was then measured by using a fluorescence plate reader at 360 nm (excitation)/465 nm (emission).

### Mitochondrial membrane potential (ΔΨm) assay

ΔΨm was analyzed by using the 5,5',6,6'-tetrachloro-l,1',3,3'-tetraethylbenzimidazol-carbocyanine iodide (JC-1) mitochondrial membrane potential assay kit (Cayman Chemical, MI, USA). Briefly, after treatment, JC-1 staining solution was added to each well and mixed gently at 37°C for 15-30 min in the dark. JC-1 assay buffer was added to each well, and plates were analyzed by using a fluorescent plate reader at excitation/emission: 535/595 nm and 485/535 nm.

### Design and synthesis of locked nucleic acid (LNA) oligonucleotides

LNA antisense oligonucleotides, are ideal whenever short or very similar sequences need to be analyzed, were designed with sequence identity to the A_2A_R mRNA sequence (LNA-A_2A_R) and provided by Exiqon (Vedbaek, Denmark). LNA-modified anti-scramble or anti-A_2A_R oligonucleotide (Exiqon) is a 16-nt gapmer whose sequence is ACGGTAGGCGTAGATG.

### Western blot

Cell lysates and tissue samples containing equal amounts of protein underwent SDS-PAGE gel, transferred onto a polyvinylidene difluoride (PVDF) membrane (Millipore, Billerica, MA, USA), and immunoblotted with a primary antibody, followed by incubation with a horseradish peroxidase (HRP)-conjugated secondary antibodies. HRP substrate peroxide solution/luminol reagents (Immobilon Western Chemiluminescent Substrate, Millipore) were added and the secondary antibody signals on membranes were visualized with the Fujifilm LAS4000 luminescent image analysis system. The following primary antibodies were used: anti-p53, anti-p21, anti-p16, anti-SIRT1, anti-caspase 3, anti-PARP, anti-A_2A_R, anti-Tuj1, anti-iNOS, anti-calretinin, and anti-NeuN (GeneTex); anti-phospho-Rb, anti-phospho-Akt, anti-Rb, and anti-Akt (Cell Signaling Technology); anti-doublecortin (DCX) and anti-ionized calcium binding adapter molecule 1 (Iba1) (Abcam); and anti-glial fibrillary acidic protein (GFAP) (Millipore). All antibodies were used at dilution 1:1,000.

### Animal grouping and drug treatment

A total of 30 young adult male C57BL/6 mice (6 weeks old) were purchased from the National Laboratory Animal Center (Taipei). After 2 weeks of acclimatization, mice were randomly divided into 5 groups (n=6 each) for treatment: young control (control), D-galactose–induced mimetic aging model (D-gal), two doses of T1-11 (1 and 10 mg/kg), and vitamin E (Vit.E; 100 mg/kg) intervention aging groups. The Vit.E group was the positive control because it has antioxidant and anti-inflammatory properties and regulates adult neurogenesis [44]. The mice of D-gal, and T1-11 and Vit.E-treated groups were subcutaneously injected with 200 mg/kg D-gal daily for 8 weeks to establish the aging model. The subsequent doses of T1-11 (1 and 10 mg/kg) and one dose of Vit.E (100 mg/kg) were administered (i.g. oral gavage) every day. The mice of the young control group were similarly administered saline (0.1 ml/kg) for 8 weeks. All animals were allowed to move, drink, and take food freely. The animals were housed in standard polypropylene cages with a wired-net top in a controlled room (temperature 23 ± 1°C, humidity 55 ± 10%, 12-hr-light-dark cycle) and were allowed free access to a standard laboratory pellet diet and water during the experiments. All animal groups (n = 30) were used for analyzing immunohistochemical staining.

### Nest building test

The nesting test was performed as described [45, 46] with modification. In brief, two nestlets (5 g) were placed into the cage at 1 hr before the dark cycle, then the nest score and the weight of unshredded nestlets were determined after overnight. Nest construction was scored on a six-point scale. The nests were scored by two independent observers blinded to the group according to the following scale: 0= undisturbed; 1= disturbed; 2= flat nest; 3= cup-shaped nest; 4= incomplete dome; 5= complete dome.

### Burrowing test

We used the burrowing test described previously [46, 47] with minor modifications. In brief, at least 2 hr before the start of the dark period, mice (not food deprived) were placed in individual polysulfone cages 42.5×26.6×18.5 cm with corncob bedding, containing a cylinder (gray plastic, 20 cm long, 7 cm diameter) filled with 230 g normal diet food pellets. The lower end was sealed, resting on the cage floor. The open end was supported about 2-2.5 cm above the floor to prevent the contents from being non-purposefully displaced. Finally, the remaining food pellets in the cylinder were weighed after 2 hr. Results show the 2-hr measurement of burrowed food pellets in the second individual test.

### Morris water maze test

During this test, mice were required to learn to navigate a circular tank of water and to locate and then climb onto a hidden platform by using the cues present in the room. Details of the experimental procedure are described in Tzeng et al. [46]. Briefly, the water maze apparatus consisted of a large stainless-steel circular pool (120 cm in diameter and 40 cm high), 40 cm deep, which was filled with water (22-25°C) to a depth of 20 cm for covering a platform (diameter 10 cm). The pool was divided virtually into four equal quadrants. The transparent platform (escape platform) was submerged 1 cm below the surface of the opaque water in a fixed location.

The spatial learning-memory test included a place navigation test and a probe test. In the place navigation test, each mouse received 4 training trials with different starting positions each day for 6 consecutive days. After climbing onto the platform, the mouse was permitted to remain there for 30 s. If the mouse failed to reach the platform within 60 s, it was gently placed on the platform and allowed to remain there for 30 s. The time taken to reach the platform (escape latency) was recorded. On day 7, the probe test without the platform was conducted with a cutoff time of 90 s. The time spent in the target quadrant that previously contained the platform and the number of platform location crossings were recorded. Data acquisition involved a computerized video imaging analysis system (Ethovision, Noldus Information Technology, Leesburg, VA, USA).

### Tumor necrosis factor (TNF) content in mice hippocampus

Concentrations of cytokines (TNF-α) were determined in serum aliquots from different groups of mice. TNF-α determination was carried out employing immunoassay and quimioluminiscence techniques using TNF-α ELISA kit (Boster biological technology Co. Ltd, CA, USA).

### Measurement of the anti-oxidation activity in mice

For biochemical analysis, tissues of each mouse including cortex and hippocampus were quickly harvested and homogenated by lysis buffer, followed it by centrifuged for 10 min at 4 °C. Serum was obtained after the blood was centrifuged at 3,000 rpm for 5 min. The activities of superoxide dismutase (SOD), catalase (CAT), and glutathione peroxidase (GSH-Px), as well as the levels of malondialdehyde (MDA) in cortex, hippocampus, and serum, were determined by the assay kits and following the manufacturers protocol (Cayman Chemicals, MI, U.S.A).

### 5-bromo-2’-deoxyuridine (BrdU) administration

After 8-week inducement, we intraperitoneally injected BrdU (Sigma, St. Louis, MO, USA) to all group mice at a dosage of 50 mg/kg in saline twice daily for seven consecutive days for examining the effects of D-gal and T1-11 treatment on the differentiation of BrdU-positive cells into mature neurons in the dentate gyrus. Animals were sacrificed after the final day of BrdU treatment for histology analysis

### Immunohistochemistry

Immunohistochemistry was performed as described previously [46]. In brief, tissue sections for detecting neurogenesis were incubated in 10 mM sodium citrate (pH 6.0) at 80 °C for 30 min, and then in 2 M HCl at 37 °C for 30 min. The sections were then incubated in blocking buffer and antibody dilution buffer with primary antibodies, including mouse monoclonal antibodies to BrdU (1:500, Santa Cruz Biotechnology) and rabbit polyclonal antibody to doublecortin (DCX, 1:500, Abcam), and corresponding secondary antibodies, as mentioned above. The number of BrdU- or DCX-positive cells in the subgranular zone (SGZ) of the dentate gyrus was quantified and revealed as the linear density of BrdU- or DCX-positive cells per millimeter SGZ.

### Ethics statement

All animal procedures and protocols were performed in accordance with *The Guide for the Care and Use of Laboratory Animals* (NIH publication, 85-23, revised 1996), and were reviewed and approved by the Animal Research Committee at National Research Institute of Chinese Medicine under IACUC protocol no: 106-417-5.

### Serum biochemical indexes

Serum was assayed for glucose (Glu), aspartate transaminase (AST), alanine transaminase (ALT), triglycerides (TG), total cholesterol (TC), blood urea nitrogen (BUN), and lactate dehydrogenase (LDH) levels by using a FUJI DRI-CHEM 3000i Automated Clinical Chemistry Analyzer (FUJI Inc., Japan).

### Statistical analysis

All analyses involved using IBM SPSS v23.0. Descriptive statistics are presented as mean ± standard error of the mean (SEM). The parametric data were analyzed by unpaired two-tailed Student’s *t* test or one-way analysis of variance (ANOVA) with post-hoc Bonferroni’s multiple comparisons tests. All calculated *p*-values were two-tailed. Statistical significance was defined at *p* < 0.05.

## Supplementary Material

Supplementary Figures

Supplementary Table 1
