# Supplementary material for: T1-11, an adenosine derivative, ameliorates aging-related behavioral physiology and senescence markers in aging mice
Source: Aging (Albany NY). 2020 Jun 5;12(11):10556–77. doi: 10.18632/aging.103279 (PMC7346012; doi:10.18632/aging.103279)
Supplement: Supplementary Figures [file aging-12-103279-s002..pdf]

SUPPLEMENTARY FIGURES

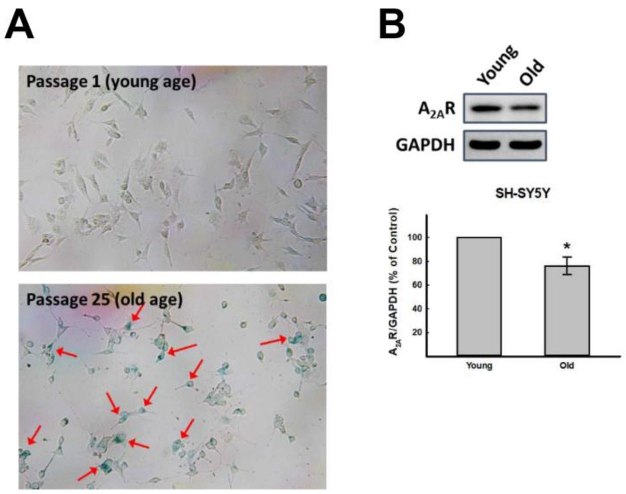

Supplementary Figure 1. Characterization of senescent SH-SY5Y cells (**A**) Micrographs of senescence-associated β-galactosidase activity in young-generation (passage 3) and senescent-generation (passage 25) SH-SY5Y cells. (**B**) Western blot analysis of levels of $A_{2A}R$ and loading control, GAPDH, in young-generation and senescent-generation SH-SY5Y cells.

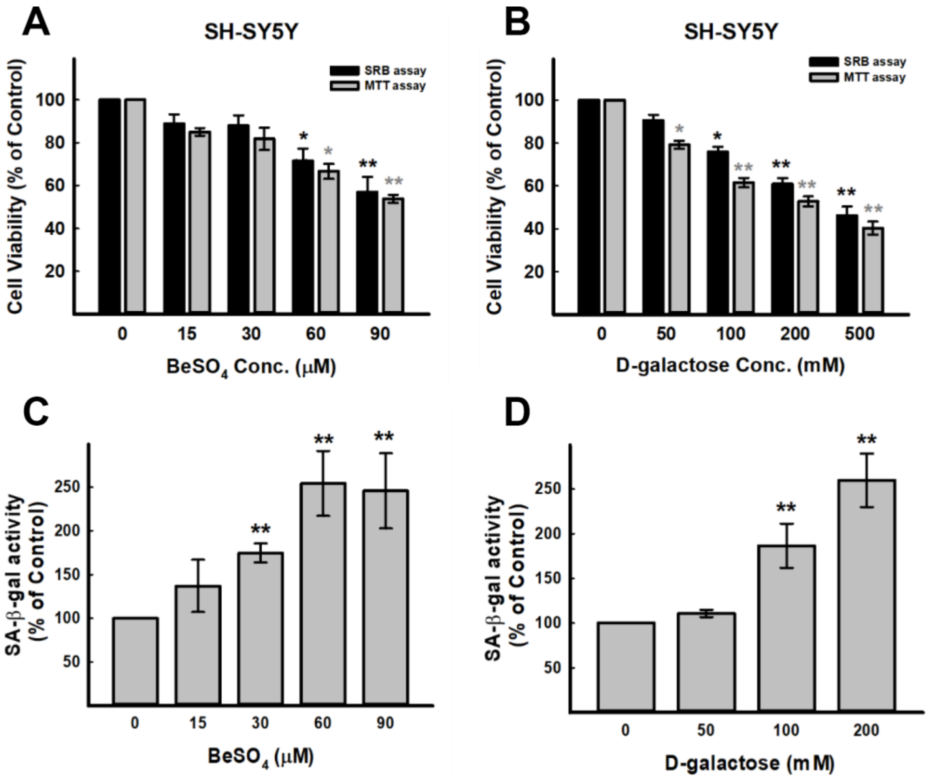

Supplementary Figure 2. Building the cellular aging model. Effect of (**A**) D-galactose and (**B**) $BeSO_4$ on cell viability. Effect of (**C**) D-galactose and (**D**) $BeSO_4$ on cellular senescence. Data are mean±SEM. *$p < 0.05$, ** $p < 0.01$ compared to control; ## $p < 0.01$ compared with D-gal treatment.

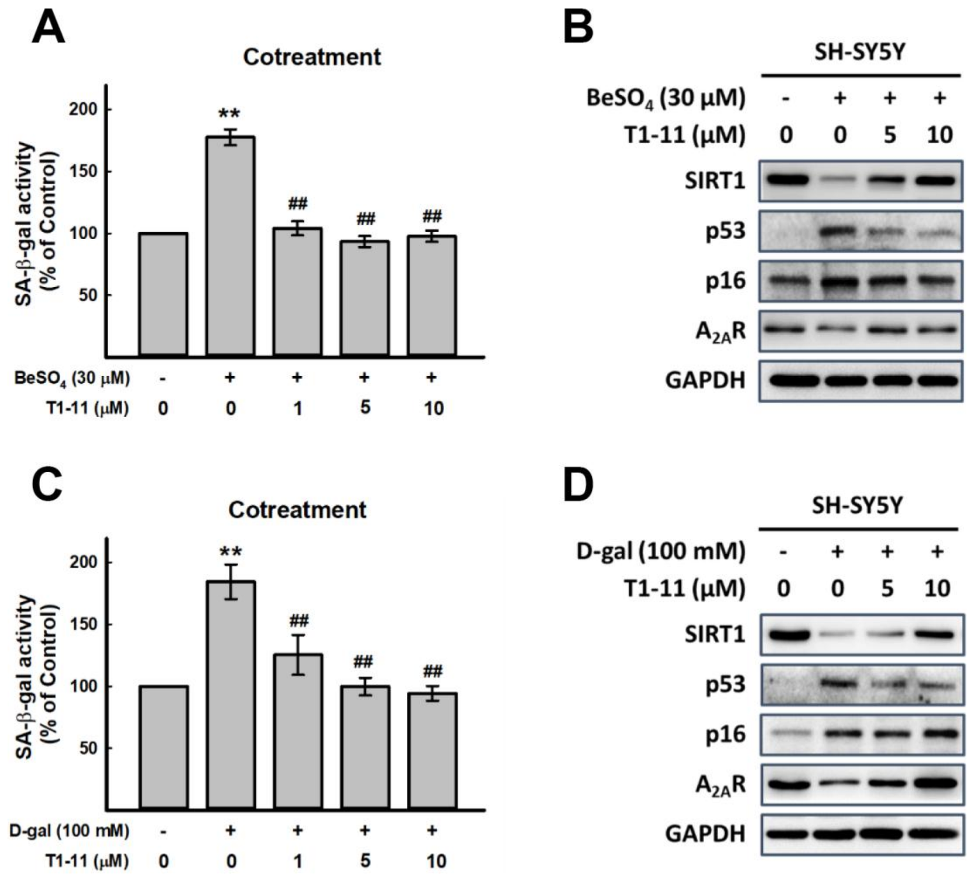

Supplementary Figure 3. Effect of T1-11 on cellular senescence after $BeSO_4$- and D-gal-induced senescence. (**A**, **C**) Effect of co-treatment of T1-11 on $BeSO_4$- and D-gal-induced cellular senescence markers, SA-β-gal activity, in SH-SY5Y cells. (**B**, **D**) Effect of co-treatment of T1-11 on $BeSO_4$- and D-gal-induced cellular senescence related molecules in SH-SY5Y cells. Data are mean±SEM from at least four independent experiments. Significant difference between control and treated cells is indicated by $^{**}p < 0.01$. Significant difference between the cells treated with D-gal or $BeSO_4$ alone and the cells treated with D-gal or $BeSO_4$ combined with T1-11 is indicated by $^{\#}p < 0.05$, $^{\#\#}p < 0.01$.

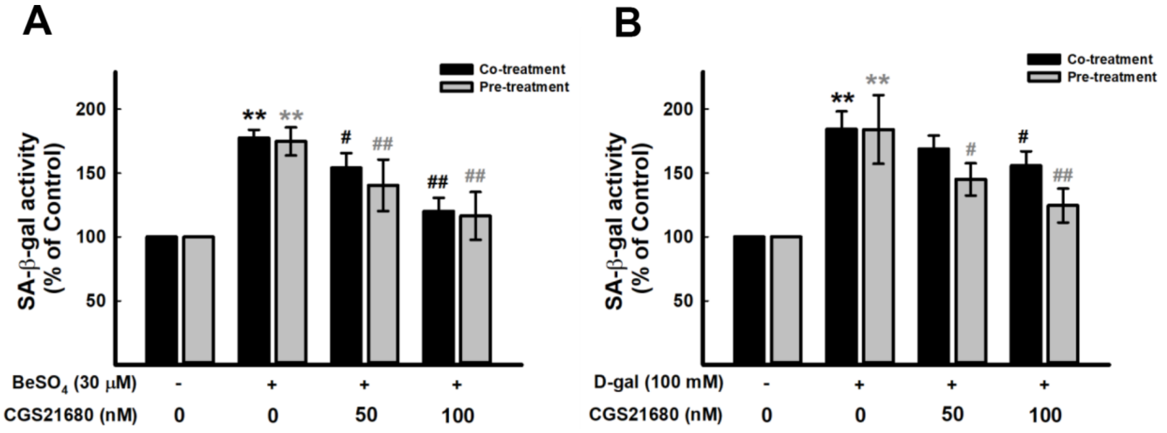

Supplementary Figure 4. Effect of CGS21680 on cellular senescence after $BeSO_4$- and D-gal-induced senescence. (**A**, **B**) Effect of co-treatment and pre-treatment of CGS21680 on $BeSO_4$- and D-gal-induced cellular senescence markers, SA-β-gal activity, in SH-SY5Y cells. Data are mean±SEM from at least three independent experiments. Significant difference between control and treated cells is indicated by $^{**}p < 0.01$. Significant difference between the cells treated with D-gal or $BeSO_4$ alone and the cells treated with D-gal or $BeSO_4$ combined with CGS21680 is indicated by $^{\#}p < 0.05$, $^{\#\#}p < 0.01$.

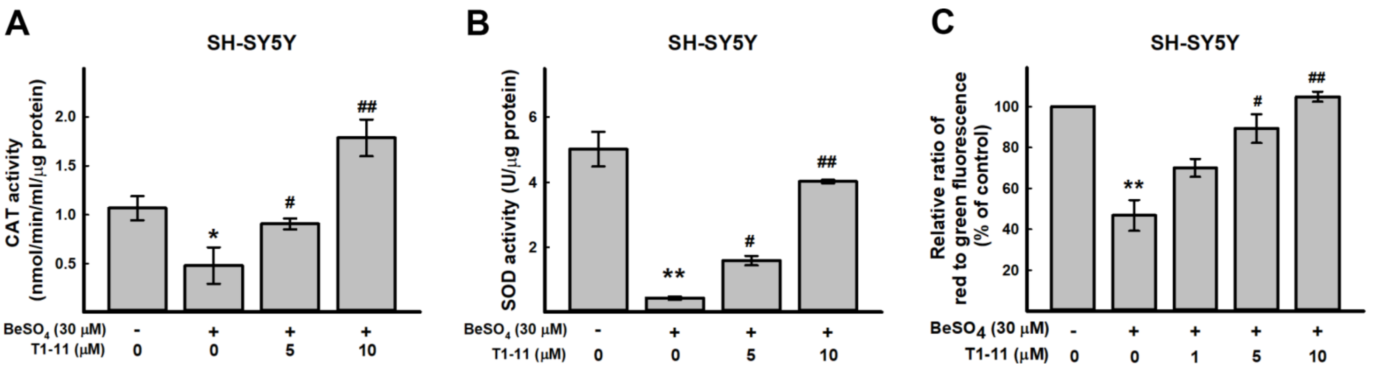

Supplementary Figure 5. Effect of T1-11 on activities of antioxidant enzymes and mitochondrial membrane potential (ΔΨm) in $BeSO_4$-induced aging SH-SY5Y cells. Effect of T1-11 on (**A**) superoxide dismutase (SOD) and (**B**) catalase (CAT) activities in SH-SY5Y cells with $BeSO_4$ treatment. (**C**) SH-SY5Y cells were pre-treated with T1-11 for 24 hr, then exposed to 30 µM $BeSO_4$ for 24 hr. Quantification of ΔΨm observed by spectrophotometry. T1-11 protects against ΔΨm damage in SH-SY5Y cells injured by $BeSO_4$. Data are mean ± SD. * $p < 0.05$, ** $p < 0.01$ compared to control; # $p < 0.05$, ## $p < 0.01$ compared with D-gal or $BeSo_4$ treated cells at the same incubation time.

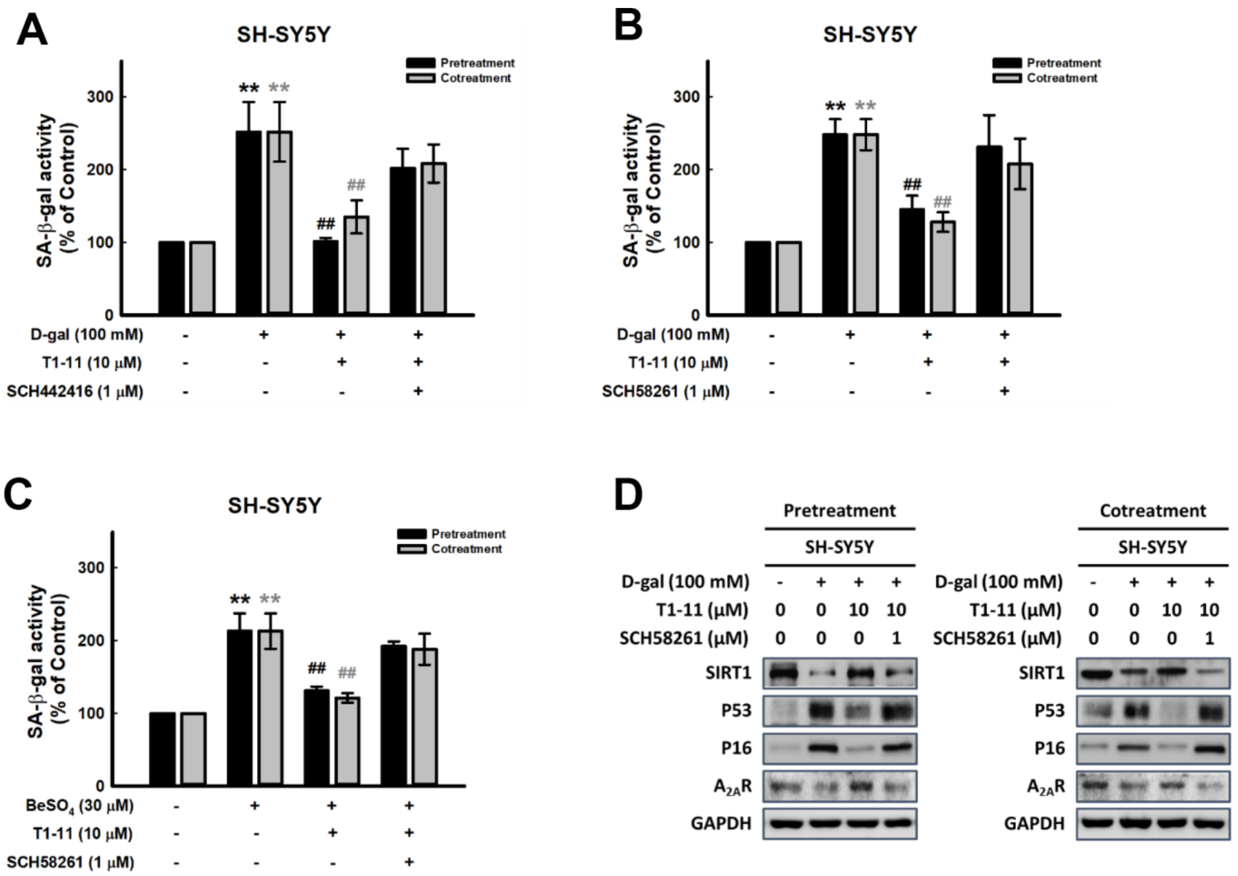

Supplementary Figure 6. Effect of combination of T1-11 and $A_{2A}R$ specific antagonists on cellular senescence after $BeSO_4$- or D-gal-induced senescence. (**A**, **B**) Effect of co-treatment or pre-treatment of T1-11 and combination of T1-11 and $A_{2A}R$ specific antagonists (SCH442416 or SCH58261) on D-gal-induced cellular senescence markers, SA-β-gal activity, in SH-SY5Y cells. (**C**) Effect of co-treatment or pre-treatment of T1-11 and combination of T1-11 and SCH58261 on $BeSO_4$-induced cellular senescence markers, SA-β-gal activity, in SH-SY5Y cells. (**D**) Effect of co-treatment or pre-treatment of T1-11 and SCH58261 on D-gal-induced cellular senescence related molecules in SH-SY5Y cells. Data are mean ± SEM from at least four independent experiments. Significant difference between control and treated cells is indicated by **$p < 0.01$. Significant difference between the cells treated with D-gal or $BeSO_4$ alone and the cells treated with D-gal or $BeSO_4$ combined with T1-11 is indicated by #$p < 0.05$, ##$p < 0.01$.

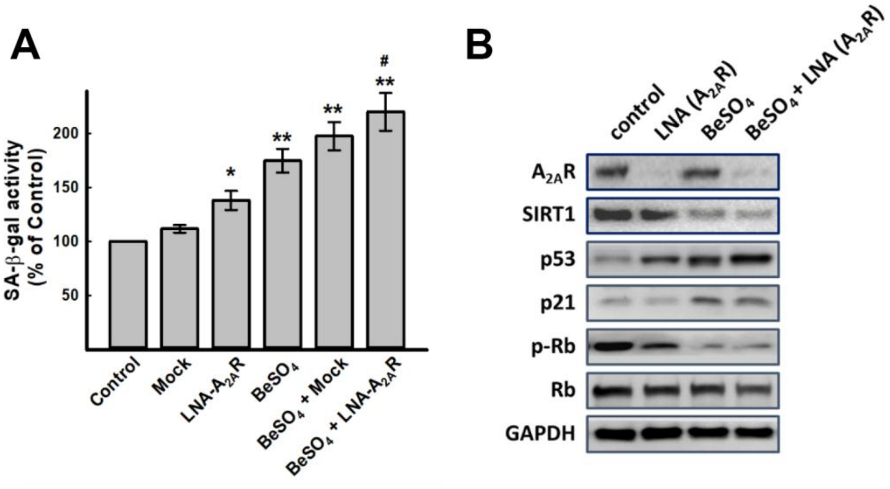

Supplementary Figure 7. Effect of $A_{2A}R$ silencing on cellular senescence in SH-SY5Y cells. Effect of the combination with LNA($A_{2A}R$) and $BeSO_4$ on cellular senescence detected by (**A**) cellular senescence assay and (**B**) western blot assay. Data are mean±SEM. *$p < 0.05$, ** $p < 0.01$ compared to control group; # $p < 0.05$, ## $p < 0.05$ compared with $BeSO_4$ treated cells.

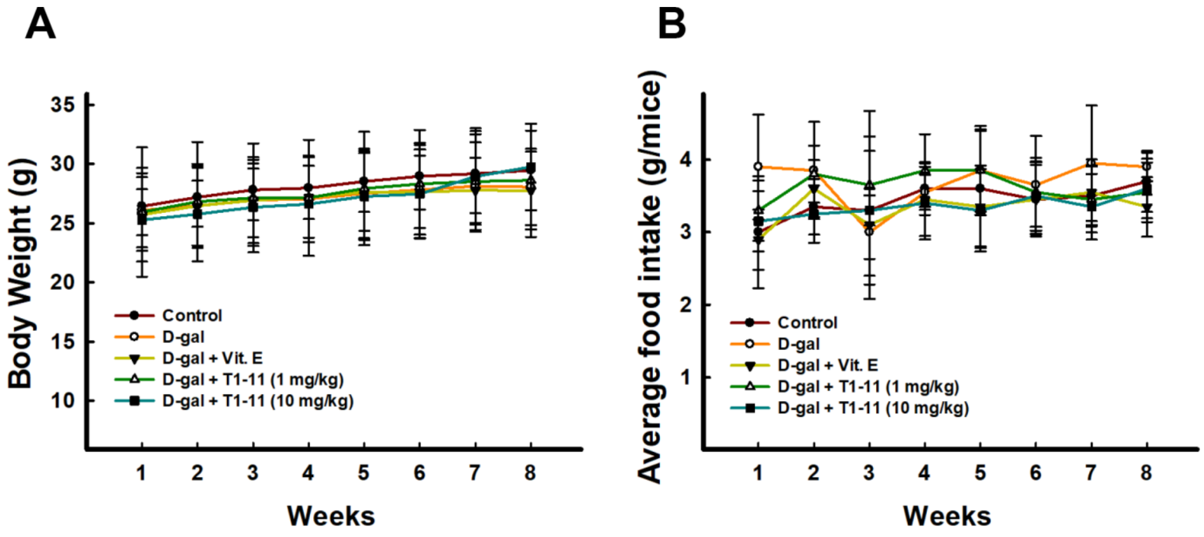

Supplementary Figure 8. Effect of T1-11 on body weight and food intake of D-gal-induced aging mice. Changes in body weight (**A**) and food intake (**B**) of the mice treated with D-gal alone and the mice treated with D-gal combined with Vit.E or T1-11 groups. All data are shown as mean ± SEM (n=6).

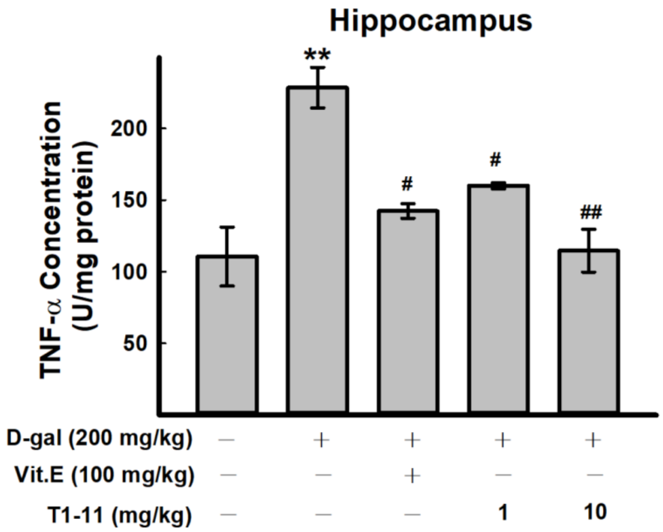

Supplementary Figure 9. Effect of T1-11 on TNF-α in hippocampus of D-gal-induced aging mice. The hippocampus tissue was collected for ELISA detection. The levels of TNF-α were shown as above. Values represent means ± SEM (n=6). *** $p < 0.001$, *$p < 0.05$ (versus the control group); ##$p < 0.01$, #$p < 0.05$ (versus the D-gal groups).

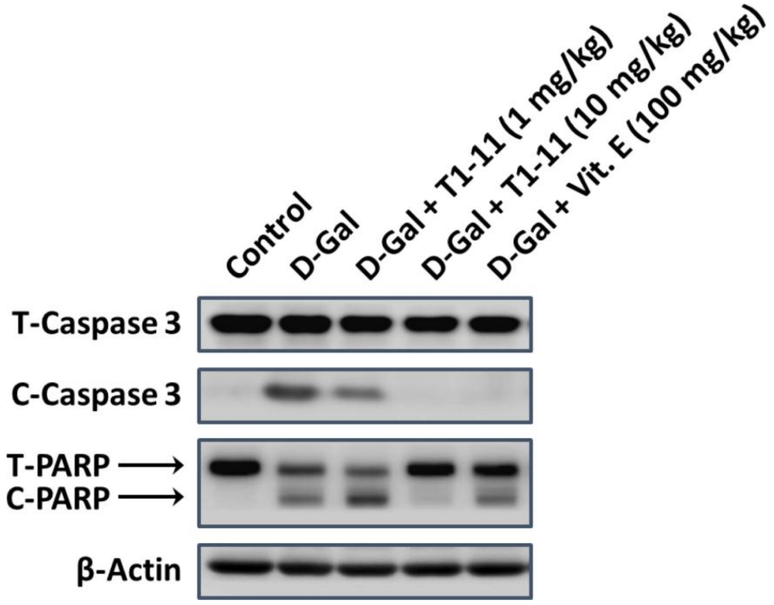

Supplementary Figure 10. T1-11 could decrease D-galactose-induced apoptosis in hippocampus of D-gal-induced aging mice. Western blot analysis of apoptosis related molecules markers in hippocampus of D-gal-induced aging mice.
