# Supplementary material for: T1-11, an adenosine derivative, ameliorates aging-related behavioral physiology and senescence markers in aging mice
Source: Aging (Albany NY). 2020 Jun 5;12(11):10556–77. doi: 10.18632/aging.103279 (PMC7346012; doi:10.18632/aging.103279)
Supplement: Supplementary Table 1 [file aging-12-103279-s001..pdf]

SUPPLEMENTARY TABLE

Supplementary Table 1. Effect of T1-11 on serum parameters.

	Glu (mg/dl)	AST (U/l)	ALT (U/l)	TG (mg/dl)	TC (mg/dl)	CRE (mg/dl)	BUN (mg/dl)	LDH (U/l)
Control	175.5 ± 36.5	45.2 ± 10.2	15.7 ± 1.9	56.5 ± 11.4	56.2 ± 5.9	0.65 ± 0.21	19.8 ± 2.4	182.8 ± 73.9
D-gal	249.7 ± 49.6**	99.0 ± 28.1**	23.8 ± 4.9*	111.0 ± 29.3*	74.0 ± 8.2*	0.28 ± 0.13*	27.6 ± 3.5**	330.3 ± 64.3**
D-gal + Vit.E	239.3 ± 27.9	62.6 ± 16.3[#]	18.4 ± 2.3	106.2 ± 18.7	72.8 ± 2.7	0.34 ± 0.19	26.5 ± 2.3	202.0 ± 44.9[##]
D-gal + T1-11[a]	215.0 ± 25.7	78.6 ± 13.6	16.0 ± 4.8	51.8 ± 23.0[#]	62.6 ± 8.6	0.32 ± 0.16	25.8 ± 2.8	282.0 ± 48.1[#]
D-gal + T1-11[b]	197.3 ± 12.3[#]	55.2 ± 12.5[##]	15.5 ± 2.9[#]	27.8 ± 14.5[##]	54.2 ± 4.7[##]	0.42 ± 0.15[#]	21.9 ± 2.5[##]	176.8 ± 51.5[##]
T1-11[b]	180.8 ± 30.4	44.7 ± 7.6	16.5 ± 2.2	44.5 ± 15.6	56.3 ± 3.6	0.81 ± 0.32	19.5 ± 2.6	181.5 ± 47.3

Glu (blood glucose); AST (aspartate transaminase); ALT (alanine transaminase); TG (triglyceride); TC (total cholesterol); CRE (creatinine); BUN (blood urea nitrogen); LDH (lactate dehydrogenase).
*$p < 0.05$, **$p < 0.01$, compared with control.
[#]$p < 0.05$, ##$p < 0.01$, compared with D-galactose group.
D-gal: D-galactose (200 mg/kg).
Vit.E: vitamin E (100 mg/kg).
[a]T1-11 (1 mg/kg).
[b]T1-11 (10 mg/kg).
